# Statement of Retraction: Long intergenic non-protein coding RNA 960 regulates cancer cell viability, migration and invasion through modulating miR-146a-5p/interleukin 1 receptor associated kinase 1 axis in pancreatic ductal adenocarcinoma

**DOI:** 10.1080/21655979.2022.2145707

**Published:** 2022-11-22

We, the Editors and Publisher of the journal *Bioengineered*, have retracted the following article:

Yaoxing Huang, Qingqing Yan, Danchun Yu, Xiaojuan Sun, Shuman Jiang, Weidong Li & Lin Jia. Long intergenic non-protein coding RNA 960 regulates cancer cell viability, migration and invasion through modulating miR-146a-5p/interleukin 1 receptor associated kinase 1 axis in pancreatic ductal adenocarcinoma. Bioengineered; 2021. 12(1): 369-381. doi: 10.1080/21655979.2020.1868742.

Since publication, significant concerns have been raised about the integrity of the data and reported results in the article. When approached for an explanation, the authors did not provide their original data or any necessary supporting information. As verifying the validity of published work is core to the integrity of the scholarly record, we are therefore retracting the article. The corresponding author listed in this publication has been informed.

We have been informed in our decision-making by our policy on publishing ethics and integrity and the COPE guidelines on retractions.

The retracted article will remain online to maintain the scholarly record, but it will be digitally watermarked on each page as ‘Retracted’.

